# Supplementary material for: A generalized Knudsen theory for gas transport with specular and diffuse reflections
Source: Nat Commun. 2023 Nov 15;14:7386. doi: 10.1038/s41467-023-43104-6 (PMC10651930; doi:10.1038/s41467-023-43104-6)
Supplement: Supplementary file 1 — Supplementary Information [file 41467_2023_43104_MOESM1_ESM.pdf]

Supplemental Material for
A generalized Knudsen theory for gas transport with specular and diffuse reflections

JianHao Qian[1], HengAn Wu[1,2,*], and FengChao Wang[1,2,†]

[1]*CAS Key Laboratory of Mechanical Behavior and Design of Materials, Department of Modern Mechanics, University of Science and Technology of China, Hefei 230027, China*

[2]*State Key Laboratory of Nonlinear Mechanics, Institute of Mechanics, Chinese Academy of Science, 15 Beisihuan West Road, Beijing 100190, China*

* Corresponding author. wuha@ustc.edu.cn

† Corresponding author. wangfc@ustc.edu.cn

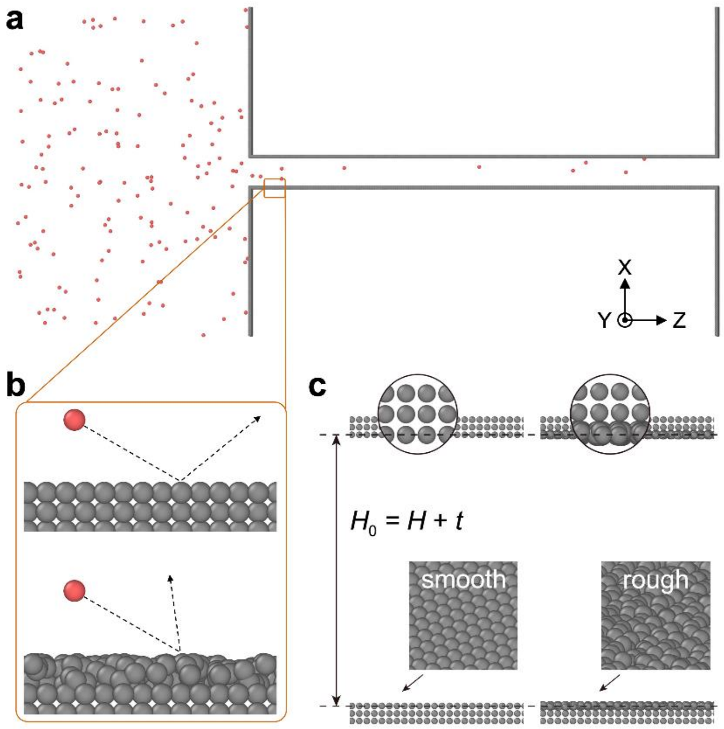

Supplementary Figure 1. Molecular dynamics (MD) simulation models. a Side view of a snapshot of MD simulations. The red and gray balls denote gas and wall atoms, respectively. **b** Illustrations of specular and diffuse reflection of gas atom from the smooth (top) and rough (bottom) channel walls. The dashed arrows represent the gas trajectories. **c** Illustration of determining the channel height for both smooth (left) and rough (right) channels.

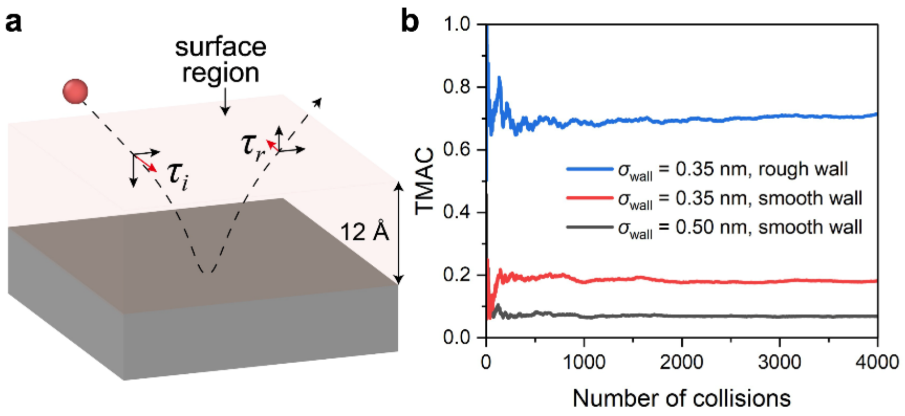

Supplementary Figure 2. Calculations on tangential momentum accommodation coefficient (TMAC). a The strategy to calculate the tangential momentum when a gas atom collides with a wall. The red ball symbolizes the gas molecule. The grey block denotes the wall. The transparent red block indicates the defined surface region with a thickness of 12 Å. The dashed arrow depicts the gas trajectory. The tangential momentum of incidence (τ_i) and reflection (τ_r) as the gas atom entered or left the surface region was calculated, as denoted by red arrows. Then the TMAC can be obtained by $(\bar{\tau}_i - \bar{\tau}_r)/\bar{\tau}_i$, in which the overline characterizes the average over a sufficient number of individual gas-wall collisions. **b** The convergence of TMAC with increasing number of collisions between argon atoms and different walls. Up to 4000 collisions were taken into account for each wall to determine the convergence value. In each collision, an argon gas atom was placed far away from the wall. Its initial velocity towards the wall obeys the Maxwell-Boltzmann distribution.

Supplementary Table 1. Parameters for channels with different surface roughness in MD simulations

Channel ID	σ (nm)	ε (meV)	d_{max} (nm)	TMAC, f
1	0.35	0.87	0	0.186
2	0.35	0.87	0.03	0.303
3	0.35	0.87	0.05	0.436
4	0.35	0.87	0.10	0.721
5	0.40	0.87	0	0.108
6	0.50	0.87	0	0.068
7	0.35	4.34	0	0.323
8	0.35	8.67	0	0.403
9	0.35	17.3	0	0.491
10	0.35	8.67	0.03	0.594
11	0.35	8.67	0.10	0.883

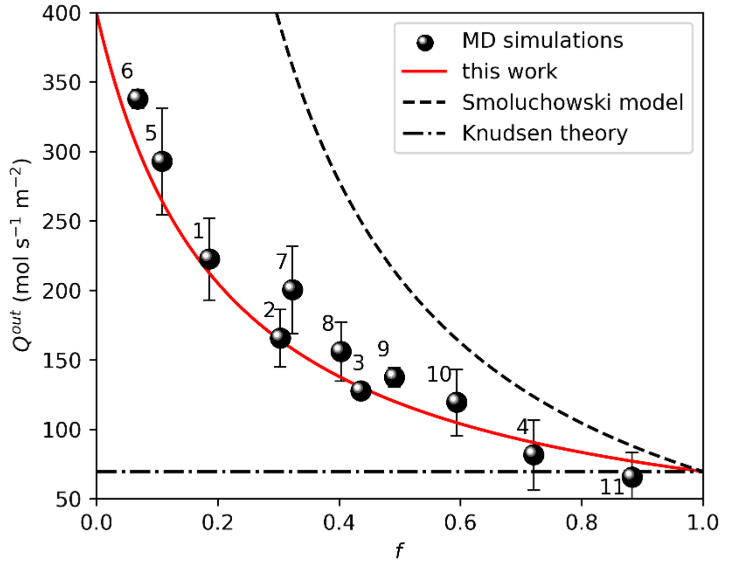

Supplementary Figure 3. Argon gas flow rates through slit channels with different surface roughness quantified using *f*. The numbers beside the data points indicate the corresponding channel ID listed in Supplementary Table 1. The slit channel has a length of 100 nm and a height of 6 nm. Error bars depict the standard deviation derived from three molecular dynamics simulations.

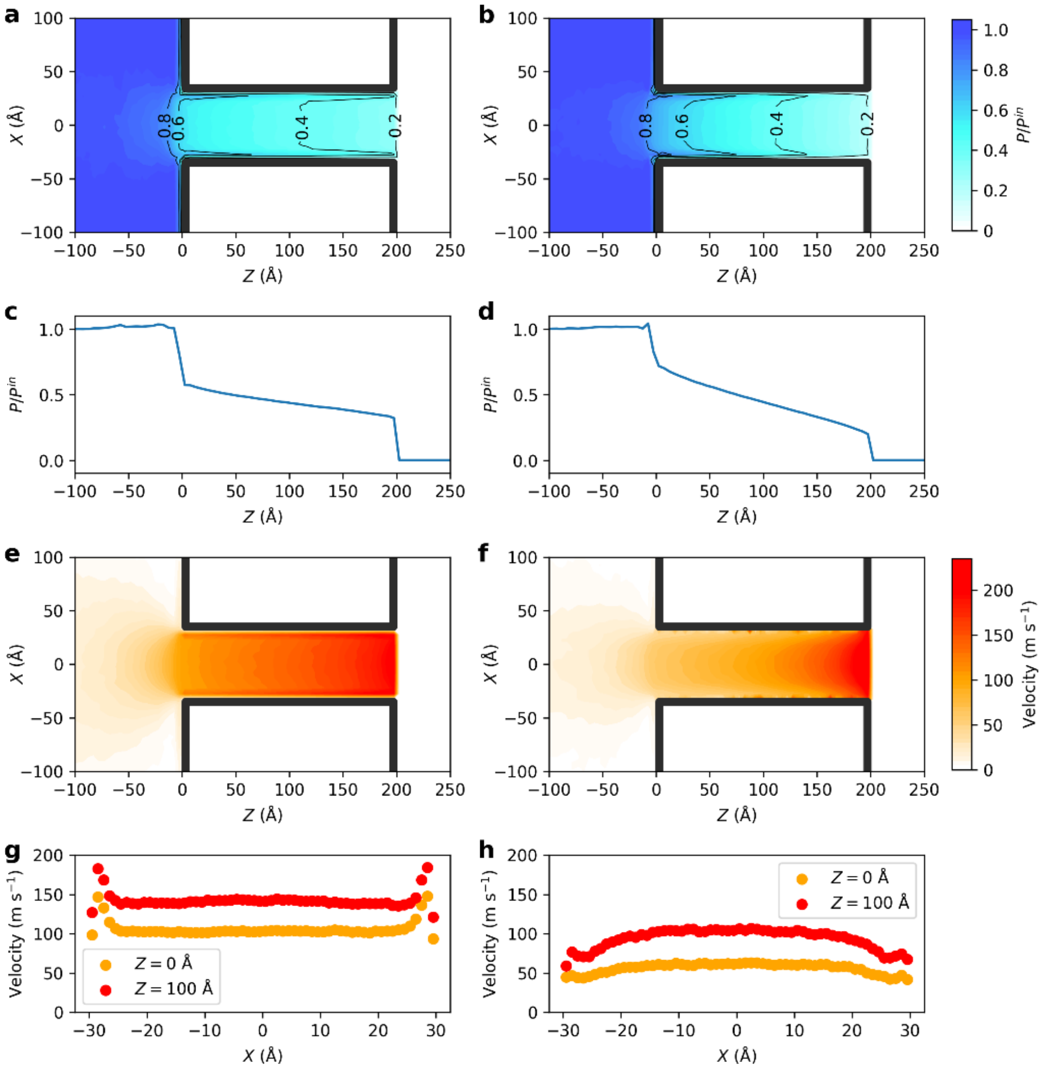

Supplementary Figure 4. Pressure distribution along the channel and velocity profiles. a Normalized pressure distribution (relative to the feed side pressure of 100 mbar) of argon gas through the slit with smooth walls ($f = 0.186$). The black bold lines represent the channel walls. **b** Same as **a**, but for a rough-walled slit ($f = 0.721$). **c** Profiles of normalized gas pressure along the axial direction of the channel with smooth walls ($f = 0.186$). **d** Same as **c**, but for a rough-walled slit ($f = 0.721$). **e** Distribution of the average gas velocity of the argon flow in a slit with smooth walls ($f = 0.186$). **f** Same as **e**, but for a rough-walled slit ($f = 0.721$). **g** Profiles of gas average velocity at the channel entrance and mid-channel with smooth walls ($f = 0.186$). **h** Same as **g**, but for a rough-walled slit ($f = 0.721$). All the slit channels have a length of 20 nm and a height of 6 nm.

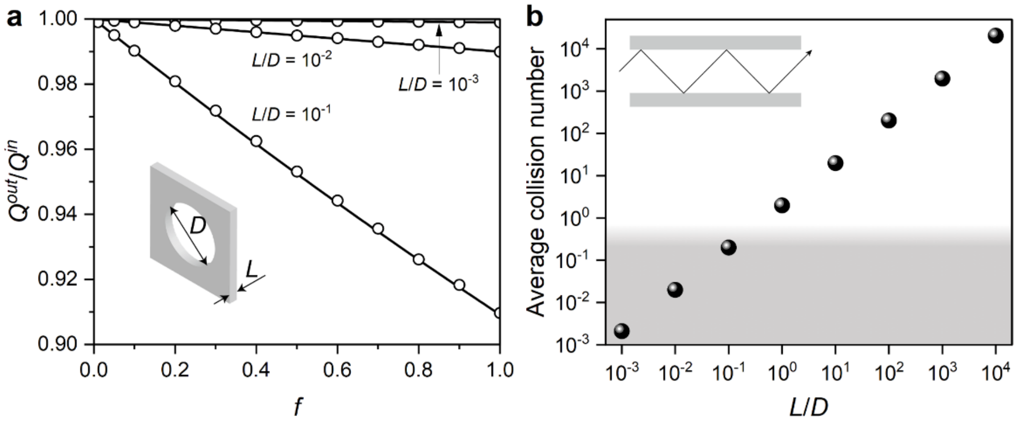

Supplementary Figure 5. Applicability of the proposed model to gas flow through an aperture. a The gas flow rate through a circular aperture as a function of the fraction of diffuse reflection, f, with varying length-to-diameter ratios, L/D, being 0.1, 0.01 and 0.001. The open circle symbols represent virtual wall simulation results. The solid lines denote results predicted by our proposed model. The inset displays the circular aperture or the pore on a thin membrane. **b** The average number of gas-wall collisions, when gas molecules pass through channels with different L/D and $f = 0$. The collisions number is recorded during simulations. Very few collisions for length-to-diameter ratio shown within the grey area and most gas molecules traverse the membrane without undergoing any collisions. Inset, a schematic of gas molecules leaving the channel after experiencing 4 collisions.

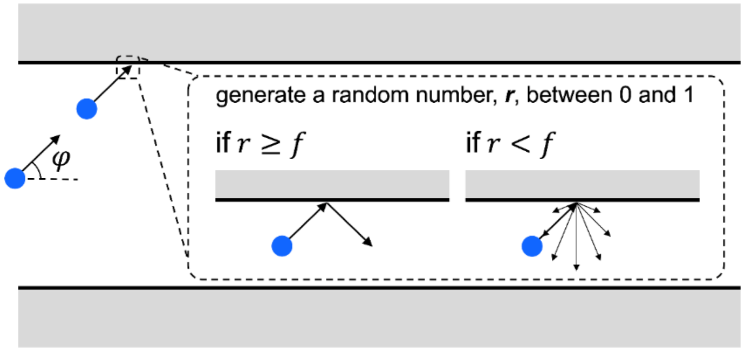

Supplementary Figure 6. Schematic of the virtual wall simulations. The gas reflection mode was determined by a random number and its comparison with a preset *f*. If the random number is smaller than *f*, the particle will be assigned a random velocity. The direction of this velocity follows the cosine law, referred to as the diffuse reflection. Otherwise, the velocity in the tangential direction remains unchanged, but its normal velocity reverses, known as the specular reflection.

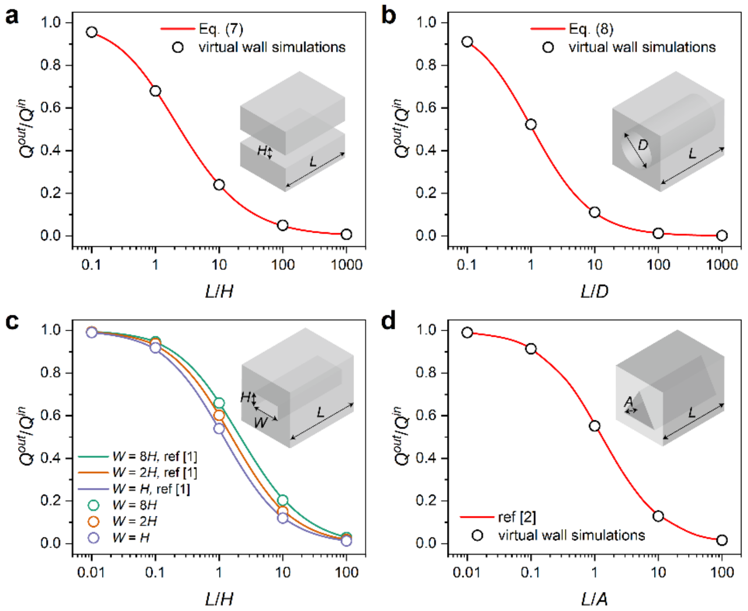

Supplementary Figure 7. The outlet-to-inlet flow rate ratio for channels with different cross-section shapes, $f = 1$. a slit, **b** circle, **c** rectangle and **d** equilateral triangle. Open circle symbols denote results calculated by the virtual wall simulations. The solid lines are results expected by the Knudsen theory. Insets give the schematic of channels and the description of channel dimensions.

Supplementary References

1. Santeler, D. J. & Boeckmann, M. D. Molecular flow transmission probabilities of rectangular tubes. *J. Vac. Sci. Technol. A* **9**, 2378–2383 (1991).
2. Li, Y., Chen, X., Wang, L., Guo, L. & Li, Y. Molecular flow transmission probabilities of any regular polygon tubes. *Vacuum* **92**, 81–84 (2013).
